# Supplementary material for: Asgard archaea capable of anaerobic hydrocarbon cycling
Source: Nat Commun. 2019 Apr 23;10:1822. doi: 10.1038/s41467-019-09364-x (PMC6478937; doi:10.1038/s41467-019-09364-x)
Supplement: Supplementary file 3 — Description of Additional Supplementary Files [file 41467_2019_9364_MOESM3_ESM.pdf]

Description of Additional Supplementary Files

File Name: Supplementary Data 1

Description: List of proteins identified in Helarchaeota genomes. Data show potential proteins analyzed in Hel_GB_A and Hel_GB_B and the corresponding gene locus for each bin. Locus ID's for both Prokka annotation and Prodigal annotation are displayed.
