# Supplementary material for: Asgard archaea capable of anaerobic hydrocarbon cycling
Source: Nat Commun. 2019 Apr 23;10:1822. doi: 10.1038/s41467-019-09364-x (PMC6478937; doi:10.1038/s41467-019-09364-x)
Supplement: Supplementary file 5 — Reporting Summary [file 41467_2019_9364_MOESM5_ESM.pdf]

nature research

Corresponding author(s): Brett Baker

Last updated by author(s): 2019/02/11

Reporting Summary

Nature Research wishes to improve the reproducibility of the work that we publish. This form provides structure for consistency and transparency in reporting. For further information on Nature Research policies, see Authors & Referees and the Editorial Policy Checklist.

Statistics

For all statistical analyses, confirm that the following items are present in the figure legend, table legend, main text, or Methods section.

n/a	Confirmed	
☒	☐	The exact sample size (*n*) for each experimental group/condition, given as a discrete number and unit of measurement
☒	☐	A statement on whether measurements were taken from distinct samples or whether the same sample was measured repeatedly
☒	☐	The statistical test(s) used AND whether they are one- or two-sided<br>*Only common tests should be described solely by name; describe more complex techniques in the Methods section.*
☒	☐	A description of all covariates tested
☒	☐	A description of any assumptions or corrections, such as tests of normality and adjustment for multiple comparisons
☒	☐	A full description of the statistical parameters including central tendency (e.g. means) or other basic estimates (e.g. regression coefficient) AND variation (e.g. standard deviation) or associated estimates of uncertainty (e.g. confidence intervals)
☒	☐	For null hypothesis testing, the test statistic (e.g. *F*, *t*, *r*) with confidence intervals, effect sizes, degrees of freedom and *P* value noted<br>*Give P values as exact values whenever suitable.*
☐	☒	For Bayesian analysis, information on the choice of priors and Markov chain Monte Carlo settings
☒	☐	For hierarchical and complex designs, identification of the appropriate level for tests and full reporting of outcomes
☒	☐	Estimates of effect sizes (e.g. Cohen's *d*, Pearson's *r*), indicating how they were calculated

Our web collection on statistics for biologists contains articles on many of the points above.

Software and code

Policy information about availability of computer code

Data collection	JGI performed read quality checks and generated a first assembly using the following methods: BBDuk adapter trimming removed known Illumina adapters. The reads were further processed using BBDuk quality filtering and trimming to remove reads quality score less than 12, containing more than three 'Ns', or with quality scores (before trimming) averaging less than 3 over the read length, or length under 51 bp after trimming. Additionally, reads matching Illumina artifacts or phiX were discarded. The remaining reads were mapped to a masked version of the human HG19 with BBMap and all hits over 93% sequence identity to the human genome were discarded. Trimmed, screened, paired-end Illumina reads were assembled using the megahit assembler using a range of Kmers. Default settings for megahit parameters were used with the following options: "--k-list 23,43,63,83,103,123". High-quality reads were mapped to the final assembly to calculate coverage information using bbmap by excluding all parameters except ambiguous=random as described by JGI.
Data analysis	The scaffolds from the assembly were binned using ESOM, MetaBAT and CONCOCT. For ESOM binning was performed on contigs with a minimum length of 2,000 bp using the K-batch algorithm for training after running the perl script esomWrapper.pl. Emerging Self-Organizing Maps (ESOM) were manually sorted and curated. Bins were extracted using getClassFasta.pl (using -loyal 51). Reference genomes were included to add genetic signatures for the assembled contigs and improve binning. For CONCOCT, Anvi'o (v2.2.2) was used as the metagenomic workflow pipeline. Coverage information was obtained by mapping all high-quality reads of each sample against the assembly of another sample using the BWA-MEM algorithm in paired-end mode (bwa-0.7.12-r1034; using default settings). The resulting sam file was sorted and converted to bam using samtools (version 0.1.19). The bam file was prepared for Anvi'o using the script anvi-init-bam and a contigs database generated using anvi-gen-contigs-database. These files were the input for anvi-profile. Generated profiles for the assemblies were combined using anvi-merge and the resulting bins summarized using anvi-summarize (-C CONCOT). If not mentioned otherwise, the scripts were used with default settings. Metabat was used as a final binning approach (v1). As described for Anvi'o the input consisted of the scaffold files (≥ 2000 bp) and the mapping files. First, each of the mapping files were summarized using jgi_summarize_bam_contig_depths and then metabat was run using the following settings: --minProb 75 --minContig 2000 --minContigByCorr 2000. Results from the three different binning tools were combined using DAS Tool (version 1.0).<br>For each of the binning tools a scaffold-to-bin list was prepared and DAS Tool run on each of the eleven scaffold files as follows: DAS_Tool.sh -i Anvio_contig_list.tsv,Metabat_contig_list.tsv,ESOM_contig_list.tsv -l Anvio,Metabat,ESOM -c scaffolds.fasta --write_bins 1. CheckM lineage_wf (v1.0.5) was run on bins generated from DAS Tool and 577 bins showed an completeness > 50% and were

characterized further. 37 Phylosift identified marker genes were used for preliminary phylogenetic identification of individual bins. Assemblies were improved by rerunning them with IDBA-UD and Metaspades. The command for IDBA was called as follows: "idba_ud -r Guay9_METAGENOME.fasta -o G9 --pre_correction --mink 75 --maxk 105 --seed_kmer 55 --num_threads 30" and Metaspades as: "metaspades.py --12 Guay16.11400.5.204846.CTCTCTA-CGTCTAA.filter-METAGENOME.fastq -o Metaspades --only-assembler --meta". Binning procedures (using scaffolds longer than 2000 bp).
Mmgenome was used to check completeness and remove contamination following the online protocol. CompareM was used to analysis differences between Helarchaeota bins and published Asgard genomes using the command python comparem aai_wf --tmp_dir tmp/ --file_ext fa -c 8 aai_compair_loki aai_compair_loki_output. The 16S rRNA gene sequences were aligned to the SILVA database (SINA v1.2.11) using the SILVA online server and Geneious (v10.1.3) was used to manually trim sequences. The cleaned alignment was used to generated a maximum-likelihood tree with RAxML as follows: "/raxmlHPC-PTHREADS-AVX -T 20 -f a -m GTRGAMMA -N autoMRE -p 12345 -x 12345 -s Nucleotide_alignment.phy -n output". We used BLASTp to identify orthologues of 56 ribosomal proteins in the two Helarchaeota bins. Individual protein datasets were aligned using mafft-linsi and ambiguously aligned positions were trimmed using BMGE (-m BLOSUM30). Maximum likelihood (ML) individual phylogenies were reconstructed using IQtree v. 1.5.5 under the LG+C20+G substitution model with 1000 ultrafast bootstraps. ML phylogenies were reconstructed using IQtree under the LG+C60+F+G+PMSF model. Bayesian phylogenies were reconstructed with Phylobayes for the dataset "without DPANN" under the LG+GTR model. Four independent Markov chain Monte Carlo chains were run for ~38,000 generations. After a burn-in of 20%, convergence was achieved for three of the chains (maxdiff < 0.29). Four independent Markov chain Monte Carlo chains were run for ~49,000 generations. After a burn-in of 20 convergence was achieved for all four the chains (maxdiff < 0.19). Gene prediction for the two Helarchaeota bins was performed using prodigal (V2.6.2) with default settings and Prokka (v1.12) with the extension '–kingdom archaea'. All the hypothetical proteins inferred in both Helarchaeaota were used as seeds against InterPro, arCOG and nr using BLAST. The annotation table from Zaremba-Niedzwiedzka, et al. 2017. was used as a basis for the comparison. Alignment was performed using Muscle and trimming used BMGE (-m BLOSUM30). IqTree was carried out using the best fit model (LG+R9). Genes were annotated by uploading the protein fasta files from both methods to KAAS (KEGG Automatic Annotation Server) for complete or draft genomes to assign orthologs. Files were run using the following settings: prokaryotic option, GhostX and bi-directional best hit (BBH). Additionally, genes were annotated by JGI-IMG to confirm hits using two independent databases. Hits of interest were confirmed using blastp on the NCBI webserver with default settings. Hits of interest were confirmed using Uniprot was used with default settings. Hydrogenases were identified by the HydDB database. A small subunit tree was generated by aligning sequences from Helarchaoeta and published sequences as classified by Vignias et al. in Geneious and running a maximum-likelihood phylogenetic tree using the command raxmlHPC-PTHREADS-AVX -T 10 -f a -m PROTGAMMAAUTO -N autoMRE -p 12345 -x 12345 -s Protein_alignment_2_masked.phy -n tree_2. The large subunit tree was made using an alignment of sequences from HydDB as previously described and the command raxmlHPC-PTHREADS-AVX -T 20 -f a -m PROTGAMMAAUTO -# 100 -p 12345 -x 12345 -s Protein_alignment_anja_edit.phy -n hydrogenase_anja_tree_2. Superfamilies were then confirmed using the online InterproScan system under default settings. The TMHMM webserver, PRED_TMR webserver and Phobius was used to identify for membrane motifs and position relative to the membrane all run under default settings.

For manuscripts utilizing custom algorithms or software that are central to the research but not yet described in published literature, software must be made available to editors/reviewers. We strongly encourage code deposition in a community repository (e.g. GitHub). See the Nature Research guidelines for submitting code & software for further information.

Data

Policy information about availability of data

All manuscripts must include a data availability statement. This statement should provide the following information, where applicable:

- Accession codes, unique identifiers, or web links for publicly available datasets
- A list of figures that have associated raw data
- A description of any restrictions on data availability

The raw reads from the metagenomes described in this study are available at JGI under the IMG genome ID 3300014911 and 3300013103 for samples 4569-2 and 4571-4, respectively. Genome sequences are available at NCBI under the accession numbers SAMN09406154 and SAMN09406174 for Hel_GB_A and Hel _GB_B respectively. Both are associated with BioProject PRJNA362212.

Field-specific reporting

Please select the one below that is the best fit for your research. If you are not sure, read the appropriate sections before making your selection.

☐ Life sciences ☐ Behavioural & social sciences ☒ Ecological, evolutionary & environmental sciences

For a reference copy of the document with all sections, see nature.com/documents/nr-reporting-summary-flat.pdf

Ecological, evolutionary & environmental sciences study design

All studies must disclose on these points even when the disclosure is negative.

Study description	Samples analyzed here are part of a study that aims to characterize the geochemical conditions and microbial community of Guaymas Basin (GB) hydrothermal vent sediments (Gulf of California, Mexico). DNA was extracted from sediment samples using the MO BIO – PowerMax Soil DNA Isolation kit and sent to the Joint Genome Institute (JGI) for sequencing. A lane of Illumina reads (HiSeq–2500 1TB, read length of 2x151 bp) were generated for samples. De novo Assembly and Binning was used to create metagenome-assembled genomes (MAGs). Phylogeney was preformed to identify genomes of interests and two novel genomes were chosen for further metabolic analysis in this study.
Research sample	The two genomic bins discussed in this paper, Hel_GB_A and Hel_GB_B, were obtained from sediment core samples collected in December 2009 on Alvin dives 4569_2 and 4571_4 respectively

Sampling strategy	Samples were taken based on distance to previously studied hydrothermal vents and the presence/abscence of a microbial mat
Data collection	Guaymas Basin sediment samples were collected from the Gulf of California (27°N0.388, 111°W24.560) at a depth of approximately 2,000 m below the water surface. Sediment cores were collected during four Alvin dives (4488, 4569, 4567, and 4571) in 2008 and 2009. Sample site photos were compiled from the Alvin frame grabber site (http://4dgeo.whoi.edu/alvin). Intact sediments were collected during Alvin dives using polycarbonate cores (45-60 cm in length, 6.25 cm interior diameter), subsampled into cm layers under N2 gas in the ship's laboratory and immediately frozen at -80°C. Eleven sediment subsamples for DNA isolation from different depth profiles yielded sufficient genomic DNA for metagenomic sequencing.
Timing and spatial scale	Sediment cores were collected during four Alvin dives (4488, 4569, 4567, and 4571) in 2008 and 2009. Data analyzed in this study was collected in December 2009 on Alvin dives 4569_2 and 4571_4. These samples were chosen based on the presence of the novel phylum in the MAGs generated by De novo assembly and binning.
Data exclusions	Other data gathered from these samples are described in Dombrowski, N., Teske, A. P. & Baker, B. J. Extensive metabolic versatility and redundancy in microbially diverse, dynamic Guaymas Basin hydrothermal sediments. Nat. Commun. In Review, (2018).
Reproducibility	Sequencing reads are available in JGI and methods provide detailed descriptions for reproducibility
Randomization	Randomization is not relevant with De novo binning and assembly methods
Blinding	Blinding is not relevant with De novo binning and assembly methods.

Did the study involve field work? ☒ Yes ☐ No

Field work, collection and transport

Field conditions	Sediment cores were collected during four Alvin dives (4488, 4569, 4567, and 4571) in 2008 and 2009.
Location	Guaymas Basin sediment samples were collected from the Gulf of California (27°N0.388, 111°W24.560) at a depth of approximately 2,000 m below the water surface.
Access and import/export	Sediment cores were collected during four Alvin dives (4488, 4569, 4567, and 4571) in 2008 and 2009. Sample site photos were compiled from the Alvin frame grabber site (http://4dgeo.whoi.edu/alvin). Intact sediments were collected during Alvin dives using polycarbonate cores (45-60 cm in length, 6.25 cm interior diameter), subsampled into cm layers under N2 gas in the ship's laboratory and immediately frozen at -80°C.
Disturbance	n/a

Reporting for specific materials, systems and methods

We require information from authors about some types of materials, experimental systems and methods used in many studies. Here, indicate whether each material, system or method listed is relevant to your study. If you are not sure if a list item applies to your research, read the appropriate section before selecting a response.

Materials & experimental systems

n/a	Involved in the study	
☒	☐	Antibodies
☒	☐	Eukaryotic cell lines
☒	☐	Palaeontology
☒	☐	Animals and other organisms
☒	☐	Human research participants
☒	☐	Clinical data

Methods

n/a	Involved in the study	
☒	☐	ChIP-seq
☒	☐	Flow cytometry
☒	☐	MRI-based neuroimaging
